# Beacon-based (bbFISH^®^) technology for rapid pathogens identification in blood cultures

**DOI:** 10.1186/1471-2180-14-99

**Published:** 2014-04-22

**Authors:** Christina Sakarikou, Martina Parisato, Giuliana Lo Cascio, Carla Fontana

**Affiliations:** 1 Department of Experimental Medicine and Surgery, “Tor Vergata” University of Rome, Via Montpellier 1, 00133 Rome, Italy; 2 Clinical Microbiology Laboratories, Foundation Polyclinic Tor Vergata, V.le Oxford 81, 00133 Rome, Italy; 3 Department of Pathology and Diagnostic, Servizio Microbiologia e Virologia-Azienda Ospedaliera Universitaria Integrata, Verona, P.le Scuro, 10-37134 Verona, Italy

**Keywords:** Sepsis, Rapid test, Beacon-based FISH, hemoFISH

## Abstract

**Background:**

Diagnosis and treatment of bloodstream infections (BSI) are often hampered by the delay in obtaining the final results of blood cultures. Rapid identification of pathogens involved in BSI is of great importance in order to improve survival of septic patients. Beacon-based fluorescent in situ hybridization (hemoFISH^®^ Gram positive and hemoFISH^®^ Gram negative test kits, miacom diagnostics GmbH Düsseldorf, Germany) accelerates the identification of most frequent bacterial pathogens of sepsis.

**Results:**

In this study a total of 558 blood culture (377 blood culture positive and 181 negative) were tested with the hemoFISH^®^ method and the results were evaluated in comparison with the traditional culture based methods. The overall sensitivity and specificity of the hemoFISH^®^ tests were 94.16% and 100%, while, the PPV and NPV were 100 and 89.16%, respectively. As the hemoFISH^®^ results were obtained within 45 mins, the time difference between the final results of the traditional culture method and the hemoFISH^®^ assay was about two days.

**Conclusions:**

Considering the good sensitivity and specificity of the hemoFISH^®^ assays as well as the significant time saving in obtaining the final results (p-value 0.0001), the introduction of the system could be rialable in the microbiology laboratories, even alongside the traditional systems.

## Background

Sepsis is a serious clinical syndrome resulting from a host’s systemic inflammatory response to infection [1]. When severe, it is associated with high mortality, greater in patients with septic shock (40-70%), than in those with sepsis alone (25-30%). The syndrome is nowadays considered as a major international health care problem [2,3]. Bloodstream infection is commonly associated with the development of sepsis and requires microbiological diagnosis usually performed by traditional culture, detection and identification of the causative pathogens of the systemic inflammatory response syndrome (SIRS) [3-5]. However, culture routinely takes several days before a positive result is available [6]. This gap between the initial clinical suspicion and the confirmation of infection by culture results could result in a poor clinical outcome of the septic patient [7,8]. The long total turnaround time (TAT) which characterizes traditional culture methods encourages clinicians in empirical antimicrobial therapy as a safety-first strategy. The delay in appropriate antimicrobial therapy is associated with increased mortality [7,8]. Therefore, there is an urgent need to introduce techniques, with a reduced TAT, which allow the clinicians to set therapeutic regimens in the earlier stages of sepsis. Molecular methods seem to be an appropriate choice, they are widely used in the diagnosis of BSIs, along side to the conventional methods. Molecular techniques are based on amplification of nucleic acids, species-specific hybridization, microarray technology and gene sequencing [9]. However, these techniques involve significantly increased cost and technical complexity, both of which are likely to hamper their adoption in the laboratory routine in the clinical setting. Fluorescent in-situ hybridization (FISH) technique is based on fluorescently labelled oligonucleotide probes complementarily binding to specific target sequences in the ribosomal RNA of bacteria, yeasts or other organisms. The most commonly used target for FISH in prokaryotes is 16S rRNA, as it contains both highly stable and variable regions. However, the 23S rRNA in prokaryotes and the 18S and 28S rRNA in eukaryotes, as well as mRNA have also been used as FISH targets [10]. Since the ribosomes are found in a large number in active multiplying bacteria, especially in the log phase of bacterial growth, the amplification step is not required. Target sequences will be presented naturally in the bacteria in a concentration high enough to enable visual detection of the specific fluorescent signal. FISH was first applied for detection of prokaryotes by environmental biologists for analysis of microbial communities. The method was soon introduced to medical microbiology and ever since used in various fields of diagnostics of human infectious diseases, with emphasis on situations when a speedy identification is crucial or the pathogen is difficult to culture: sepsis, meningitis, endocarditis, respiratory tract infections, especially those of cystic fibrosis patients, screening for intrapartum *Streptococcus agalactiae* carriage, diagnosis of zoonotic infections such as those caused by *Brucella* and *Francisella*[11-17]. Miacom^®^ diagnostics GmbH has combined the classical FISH technology with the usage of fluorescently labelled DNA-molecular beacons as probes, making it an easy procedure known as the beacon-based FISH (bbFISH^®^) technology [18]. It is now possible, for the first time, to use specific probes against a wide variety of clinically relevant bacteria working directly on blood culture. The probes enter the cells, hybridize to their specific targets, making the cells visible using a fluorescence microscope. In order to assess the possible benefits of the introduction of such technology into the laboratory routine, we evaluated in the present study the performance of the bbFISH^®^ (hemoFISH^®^ Gram positive and hemoFISH^®^ Gram negative) in comparison to the conventional culture of bacteria from positive blood culture vials in febrile patients. The study was conducted independently in two Italian centers: Polyclinic of Tor Vergata in Rome and Polyclinic Ospedale G.B. Rossi in Verona. We have also examined the hemoFISH^®^ test and the conventional identification assay’s total turnaround time (TAT) performance.

## Results

### Blood culture results

In this study 558 consecutive samples were tested: 377 positive and 181 negative. The Hospital of Verona processed 243 blood culture (88 negative and 155 positive) while the Hospital in Rome analysed a total of 315 blood cultures (93 negative and 222 positive). 393 were the isolates (239 Gram-positive and 153 Gram-negative and one yeast) identified by conventional system (Vitek 2 System), including those from 16 mixed blood cultures (those which contain two isolates).

### hemoFISH^®^ performances

The test works equally well in both centers being the overall performances substantially similar. The hemoFISH^®^ test correctly identified 364/393 isolates, showing an overall agreement of 92.6% with the culture method. If the performances were considered referred to the specimens (not the isolates) 355/377 positive specimens were identified by hemoFISH^®^ (94.16%). The sensitivity, the specificity the PPV and NPV were 94.16, 100, 100 and 89.16, respectively.

The individual Kit performances were as follows:

The hemoFISH^®^Gram negative panel correctly identified 143/153 (93.6%) of the Gram-negative for which a specific probe was present in the Kit (59 *Escherichia coli*, 26 *Klebsiella.pneumoniae*, 4 *Proteus mirabilis*, 2 *Proteus vulgaris*, 4 *Salmonella* spp, 5 *Serratia marcescens*, 14 *Pseudomonas aeruginosa*, 12 *Acinetobacter* spp, 4 *Stenotrophomonas maltophilia* and 1 *Haemophilus influenzae*) (Table 1). Two *K.pneumoniae* and one *P.mirabilis* were identified only at the genus level as *Enterobacteriaceae s*pp (Table 1). Other Gram-negatives, for which there were no specific probes on the panel, yet belonging to the *Enterobacteriaceae* group, such as *Klebsiella oxytoca*, *Enterobacter aerogenes* and *Enterobacter cloacae*, were correctly identified as *Enterobacteriaceae* spp. One *Pasteurella multocida* (for which no specific probe was present on the hemoFISH Gram negative panel) was misidentified as *Enterobacteriaceae* spp (Table 1).

**Table 1:** hemoFISH Gram positive and Gram negative panels performances in identifying Gram-negative and Gram-positive in comparison with Vitek 2 system

**Panel**	**Species**	**Strains identified using Vitek 2 system**	**Strains identified using bbFISH**	**bbFISH global percentage of identification bbFISH (%)**	**Strains misidentified by bbFISH**	**Strains not identified by bbFISH**
hemoFISH Gram positive	*S.epidermidis* and other *CoNS*^*#*^	131	130&	221/239 (92.5%)		1
	*S.aureus*	16	16			
	*Streptococcus spp*	27	27^			
	*S.pneumoniae*	5	3^			2
	*S.pyogenes*	1	1			
	*E.faecalis*	19	19			
	*E.faecium*	22	22			
	*E.gallinarum*	1	0		1°	
	*E.raffinosus*	2	0			2
	*M.luteus*	4			2&	2
	*M.lylae*	4	0			4
	*Corynebacterium* spp.	2	0			2
	*Bacillus* spp*.*	2	0			2
	*C.perfringens*	3	3			
	*C.albicans*	1				1§
hemoFISH Gram negative	*E.coli*	59	59	143/153 (93.5%)		
	*K.pneumoniae*	26	24		2*	
	*K.oxytoca*	5	5*			
	*E.aerogenes*	4	4*			
	*E.cloacae*	5	5*			
	*P.mirabilis*	5	4*		1*	
	*P.vulgaris*	2	2			
	*S.enterica*	4	4^a^			
	*S.marcescens*	5	5			
	*P.multocida*	1			1	
	*P.aeruginosa*	14	14			
	*A.baumannii*	11	11^b^			
	*A.lwoflii*	1	1^b^			
	*S.maltophilia*	4	4			
	*B.cepacia*	2	0			2
	*A.veronii*	1			1°	
	*H.influenzae*	1	1			
	*R.radiobacter*	1	0			1
	*B.fragilis*	2	0			2
	Total	393	364		8	21

CoNS^#^ = *Staphylococcus* coagulase negative, namely: *S.capitis*, *S.hominis*, *S.haemolyticus*, *S.warneri*, *S.auricolaris*, *S.saccarolyticus*, *S.cohnii*; & = *Staphylococcus* spp; ^ = *Streptococcus* spp; ° misidentified as *E.faecium*; § aspecific signal on green channel (eubacterial probe); * = *Enterobacteriaceae* spp; ^a^ = *Salmonella* spp; ^b^*Acinetobacter* spp; c Streptococcus spp, namely (*S.bovis*, *S.oralis*, *S.gallolyticus* and *S.gordonii*).

Eleven *Acinetobacter baumannii* and one *Acinetobacter lwoflii* were identified as *Acinetobacter* spp. A misidentification was assigned to *Aeromonas veronii*, which was improperly identified as *Enterobacteriaceae* spp (Table 1).

Five specimens (2 *Bacteroides fragilis*, 2 *Burkholderia cepacia* and 1 *Rhizobiom radiobacter*) were identified by the traditional method, but they only gave a signal with the positive control using the miacom test (Table 1). No probes for these species are included in the test panel so these results are conform with miacom’s claims.

The hemoFISH^®^Gram positive panel correctly identified 221/239 Gram-positive isolates (92.5%) (Table 1). Particularly, a total of 130 coagulase negative staphylococci were identified as *Staphylococcus* spp (the staphylococci identification obtained using Vitek 2 system were: 70 *Staphylococcus epidermidis*, 23 *Staphylococcus hominis*, 22 *Staphylococcus haemolyticus*, 4 *Staphylococcus warneri*, 8 *Staphylococcus capitis*, 1 *Staphylococcus auricolaris*, 1 *Staphylococcus saccharolyticus*, 1 *Staphylococcus saprophyticus*) while one sample positive for *Staphylococcus cohnii* was not identified. 16 samples, positive per *Staphylococcus aureus*, were correctly identified (Table 1).

Looking at the streptococci, 30/32 samples were correctly identified as *Streptococcus* spp (19 *Streptococcus mitis*, 1 *Streptococcus bovis*, 2 *Streptococcus oralis*, 4 *Streptococcus gallolyticus* and 1 *Streptococcus gordoni*), while among 5 specimens positive for *Streptococcus pneumoniae*, 3 were identified as *Streptococcus* spp (albeit no signal was evidenced with specific probe in *S.pneumonie* well) and 2 were not identified (only the signal with the eubacterial probe was recorded) (Table 1).

Enterococci were detected in a total of 41/44 specimens, two *Enterococcus raffinosus* were not identified and one *Enterococcus gallinarum* was misidentified by hemoFISH as *Enterococcus faecium* (Vitek 2 system identified: 19 *Enterococcus faecalis*, 22 *E.faecium*, 2 *E. raffinosus* and one *E.gallinarum*) (Table 1).

Eight specimens resulted positive for *Microcococcus* spp, namely 4 *Micrococcus luteus* and 4 *Micrococcus lylae*, of these, two (those positive for *M.luteus*) gave a positive fluorescent signal on the *Staphylococcus* spp well (recorded as misidentifications), the remaining 6 were not identified (Table 1).

Among the Gram-positive bacilli: two *Corynebaterium* spp and two *Bacillus* spp were identified in four different specimens by Vitek 2 (one *Corynebacterium amycolatum*, one *Corynebacterium* spp, one *Bacillus cereus* and one *Bacillus* spp). Identification by hemoFISH^®^ failed for all of them (neither the signal for the positive control was detected). While the hemoFISH^®^ correctly identified three *Clostridium perfringens* (Table 1).

One sample containing *Candida* did not yield a specific signal with any of the hemoFISH^®^ probes but was clearly visible via auto fluorescent signals on all fields.

A total of 29 specimens were not identified (21 strains) or misidentified (8 strains) by the hemoFISH^®^ test (29/393; 7.4%). The global performances recorded with the hemoFISH panels, in comparison with those identified by Vitek 2 system, are summarized in the Table 1.

The overall concordance between traditional culture and hemoFISH^®^ for the negative samples was 100%, no fluorescent specific signal was recorded on 181 negative blood cultures processed.

The average TAT from the hemoFISH^®^ system and the one obtained using traditional culture methods as well as the Δ (means the difference in time to achieve a final result) between the two system in both hospitals are consistently different as reported in Table 2. The results were available sooner using the hemoFISH^®^ assay (mean value 5.2) compared to the conventional assay (mean value 43.65) expressed also by a p value of 0.001 (Table 2). The Verona data was obtained calculating the work-flow on 5 days open laboratory. From all blood cultures, the growth of microorganisms was obtained after an incubation of 18-24 h and identification to the family, genus or species level was achieved after another day, except for 16 samples, which contained more than a microorganism and subcultures were required with a delay of one more day. For this reason, the average TAT obtained using traditional culture methods is 43.65 h. hemoFISH^®^ was performed in the same blood cultures, with an average TAT of 5.2 h. The Δ TAT between the two systems is 38.45 h, with a hemoFISH^®^ time savings of two days (compared with conventional laboratory identification). hemoFISH^®^ provides a same-day identification of the majority of microorganisms and the turnaround time is considerably lower than microbiological culture.

**Table 2:** The average time in obtaining results (express in TAT) of bbFISH^®^ versus traditional culture methods in and within the two hospitals

**Turn around time expressed in hours**	**Hospital of Rome**	**Hospital of Verona**	**Mean value between the two hospitals**
Average TAT bbFish^®^ (h)	8.9 (range 30 min-17,2H)	1.5 (range 30 min-150 min)	5.2
Average TAT of traditional culture method (h)	38.8	48.5	43.65
Two tailed p-value	0.0001		
Δ (earlier diagnosis) (h)	29.9	47.0	38.45

Δ = means the difference in time to achieve a final result.

## Discussion

BSI, is a serious and life-threatening condition, rapid diagnosis of BSI and identification of the pathogenic microorganisms are needed to improve the patient outcome [5,8]. Blood culture is still currently considered the “gold standard” in BSI diagnosis [8]. However, culture assays require a long time to achieve a final result [19]. On the contrary examination of positive blood cultures with specific molecular techniques based on the microscopic morphology of the detected microorganisms enables rapid and specific determination of sepsis pathogens, enhancing early adequate therapy and improving prognosis of the patients [18-20]. A timely reporting of results of a Gram stain of blood cultures to the physician already showed a decrease in mortality [20]. If the communication of a Gram stain result is combined with a presumptive diagnosis of the pathogens involved in BSI the clinician could more appropriately target the therapy. To achieve this we find plausible to put the FISH methodologies into a routine use in our laboratories. The results of our work, aimed at the evaluation of the bbFISH technology in comparison with the traditional culture techniques, confirm the diagnostic usefulness of this system. This test presents not only an excellent sensitivity and specificity, but also a greatly reduced TAT. Laboratory TAT is a reliable performance indicator, which measures the laboratory’s efficiency in producing its results [21-23]. The TAT is commonly defined as the time elapsed between ordering a laboratory test and the reporting of the results. In this study, the TAT was specified as the time lapse from when the blood culture flagged positive in the BacT/ALERT 3D^®^ system to when the final verification of the result was reported (either by the identification of the microorganisms using the hemoFISH^®^ assay or the conventional culture assay), this just to underline the advantage in using rapid detection assays compared to traditional systems, but avoiding any other interfering parameters not strictly imputable to the laboratory work flow. Our findings also underline how different workflows in microbiology laboratory are and how these can affect the TAT. The delay caused in TAT is primarily due to the pre- and post-analytical phases. The most common reasons for this delay were found to be the order processing time, the laboratory excessive queue and the instruments times [22,23]. A huge impact on TAT, particularly in analytical phase, was also due to the choice of laboratory procedures. Recently, many publications have underlined the usefulness of “rapid methods” either PCR-based or those using the newly introduced technology of matrix-assisted laser desorption/ionization time-of-flight mass spectrometry MALDI-TOF (MS) in diagnosing blood stream infections [24-26]. Moreover, delays in the reporting the tests results were generally linked to the practice of interrupting the workflow over the weekend and during the holidays. Our study, in fact, showed that the main impact in reducing the TAT is indeed in the laboratory itself, where these interruptions were longer (Verona Hospital than the Rome Hospital). No less important is the presence of skilled personal in the laboratory and their impact on reporting time, as demonstrated by the TAT recorded in the hospital of Rome. This laboratory realistically reported the timing by performing hemoFISH^®^ tests even with those specimens processed in delay, due to the lack of personnel in the laboratory (i.e. on Saturday afternoons and Sundays). This fact has had a heavy impact on the observed average TAT (8.9 vs 1.5). Faster TAT is universally seen as desirable, as the more timely and rapidly a testing is performed, the more efficient and effective will be the treatment [22,27,28]. This in turn can save not only time and money for the patient and the hospital, but more importantly it can save lives, reduce patient morbidity and help reducing the further increase of antibiotic resistance as well as a long stay at the hospital [19,20]. Thus system such as bbFISH technology which is easy to use, with a reduced TAT and which does not require sophisticated and expensive equipments (as MALDI TOF needs instead to) could be placed in a profitable manner, especially in small laboratories as well as in the hub laboratories which receive samples from other hospitals (in the latter in order to recover the time lost in the specimens transportation). The only improvement we can auspicate is the implementation of the bbFISH panels by the adding of other specific probes on the slide (such as those for identifications of *Corynebacterium*, Gram-negative anaerobe and *Microcococcus* spp) in order to reduce the number of pathogens not identified by the system. Considering our 29 strains (ten Gram-negative and 18 Gram-positive bacteria and one yeast) for which the bbFISH failed the identification, we have observed that most of them were not identified because of the absence of a specific probe (this is true for Gram-positive: *Bacillus* spp., *Corynebacterium* spp. and *Micrococcus* spp., but also for *Bacteroides* spp, *Rhizobium* spp, *A.veronii* and *P.multocida* among the Gram-negative). The remaining (two *S.pneumoniae*, one *S.cohnii*, two *E.raffinosus* two *K.pneumoniae* and one *P.mirabilis*) could be easily explained either by the sensitivity of the system or by a possible misinterpretation of the reader and finally we cannot exclude the possibility of a technical error in preparing the slide. In order to determine if the probes did really miss the corresponding bacteria or if the procedure failed for some other reason, bacteria would have to be retested from pure culture. Unfortunately at the time of data analysis, when these discrepancies were evidencied, we could have not done it anymore because the isolates had been discarded.

## Conclusions

The bbFISH technology is a new successful molecular assay, supplementing traditional approaches, speeding up the diagnosis of bloodstream infections and identifying the majority of most important sepsis pathogens. This assay has the potential to provide timely and cost-effective information on infection status, thus allowing clinicians to make more informed decisions on appropriate antibiotic therapy at an earlier stage than is possible with culture-based approaches. Complications can be avoided and hospital stay may so be reduced. The bbFISH technology can be a good choice, in order to reduce the analytical phase of TAT, in those laboratories in which, due to the high cost, technologies such as PCR and MALDI TOF cannot be introduced.

## Methods

### Specimens

A total of 558 blood cultures from different patients were included in this study. 299 were the samples processed and recorded by the Microbiology laboratory of Foundation Polyclinic of Tor Vergata and 259 by the Microbiology laboratory of Policlinico G.B.Rossi- Azienda Ospedaliera Universitaria Integrata-Verona. Both are teaching hospitals in Rome and Verona, Italy, respectively. The blood cultures included in the study were those consecutively collected and delivered in each hospital in a three months period. The blood specimens were collected in BacT/ALERT FN Plus and BacT/ALERT FA Plus vials, which were incubated in the BacT/ALERT^®^ 3D system, (bioMérieux; Marcy l’Etoile, France).

### Hospital workflow

The Verona hospital microbiology laboratory is a 5 days open laboratory, meaning that laboratory workflow is fully covered by a microbiologist from 8.00 a.m. to 3.00 p.m., Monday to Friday, but it is off duty on Saturday afternoon and on Sunday. While, the Rome laboratory has a working time divided on 7 days, from 7.30 am to 8.00 pm, but the microbiologist, on Saturday afternoon and on Sunday, is not present.

### Traditional routine methods on positive blood culture vials

The Bact/Alert 3D^®^ (bioMerieux) system was used for blood culturing. A minimum of two culture vials per patient, one aerobic and one anaerobic, were filled directly with blood according to the manufacturer instructions. Growth of microorganisms was detected by the instrument. Cultures were continued for 5 days. When blood culture vials flagged positive, some microliters from the vial were aliquoted aseptically for light microscopy. Gram stain was performed using Previ Color (bioMérieux) according to the instructions of the manufacturer and for culturing on a variety of agar plates for different growth requirements (Agar Chocolate, Columbia supplemented with 5% of sheep blood and Schaedler agar incubated under aerobic, micro-aerobic and anaerobic condition respectively) and further identified using the VITEK 2^®^ system (bioMerieux,). The cultivation and identification was performed by the same trained individuals.

### Beacon-based fluorescent in-situ hybridization (hemoFISH^®^)

Miacom’s molecular probes consist of a DNA sequence folded into a hairpin-like structure that is linked to a fluorophore on the 5′ end and to a quencher on the 3′ end. Such probes are also referred to as molecular beacons. The DNA sequence is complementary to a rRNA counterpart that is unique to the family, genus or species level of a certain organism. Because each bacterial cell includes more than 10,000 copies of rRNA, no amplification step is necessary [29]. Each rRNA copy with a bound beacon contributes to a fluorescent signal and the cell can be detected as a shining object under a fluorescence microscope. In addition to the fluorescent signal the cells morphology can be examined to confirm the result. Miacom’s hemoFISH^®^ Gram positive and hemoFISH^®^ Gram negative panels were used to perform the assay. Tests were run as soon as possible after the blood culture vial turned positive and not later than 24 hours. On positive blood cultures, dependent on the Gram strain result, either a Gram negative (hemoFISH^®^ Gram negative panel) or a Gram positive panel (hemoFISH^®^ Gram positive panel) was used. Negative blood cultures were processed using both kits (the test kits used for these studies were kindly supplied by miacom diagnostics GmbH, Düsseldorf, Germany). Briefly, an aliquot from a positive/negative blood culture vial (which was either the aerobic or the anaerobic vial but the first which turned positive) was diluted in Clinical Sample Buffer and applied, dried and fixed onto an 8 field glass slides placed on a hotplate pre-warmed to 52+/-1°C. Permeabilization of bacteria including treatment with enzymatic Lysis Solution at 52+/-1°C followed by an incubation in ethanol. Hybridization with DNA-molecular beacon probes was carried out in a hotplate hybridization chamber at 52+/-1°C followed by an incubation in a Stop Solution bath for 1 minute. This step ensures that all unbound beacons are pushed back into the closed conformation. Slides were dried, covered with mounting medium and evaluated under a fluorescence microscope. Two filter sets are required. One detects the probes labeled with ATTO550 (red channel, absorption max 554 nm/emission max 576 nm), the other one those labeled with FAM (green channel, absorption max 494 nm/emission max 520 nm). Total turn-around time of the hemoFISH^®^ assay was approximately 45 minutes (15 min of hands-on time plus the time required for microscopic observation). The list of fluorescently labeled probes used for the strain identification is the following: *Enterobacteriaceae* spp., *E.coli*, *K.pneumoniae*, *S.marcescens*, *P.mirabilis*, *P.vulgaris*, *Salmonella* spp., *P.aeruginosa*, *Acinetobacter* spp., *S.maltophilia*, *H.influenzae* (for the hemoFISH G(-) Panel) and *Staphylococcus* spp., *S.aureus*, *Streptococcus* spp., *S.pneumoniae*, *S.pyogenes*, *S.agalactiae*, *C.perfringens*, *E.faecium*, *E.faecalis* (for the hemoFISH G(+) Panel).

The first field of the slide serves as an intrinsic control of the procedure. It contains a probe that detects most Eubacteria, giving a positive signal only in the red channel. When turning to the green channel, no fluorescence should be visible. On the remaining fields, there might be pairs of probes, labeled either with FAM or ATTO 550, giving either a red or a green fluorescent signal when the specific target is encountered. If a specific target is not encountered, the unbound probes are pushed back into the initial closed conformation and no fluorescent signal is generated. Due to the use of molecular beacons, the washing step, known to be a critical and error-prone step during the FISH procedure, can be omitted.

### Statistical analysis

For database processing, data from BacT/ALERT 3D^®^ and VITEK 2^®^ system were downloaded as text files into Microsoft Excel with subsequent transfer of it into a Microsoft Access database for analysis. Final tabulation of TAT was performed using Access with report generation, including graphs, created in Excel. The comparisons between the two techniques are expressed as proportions. Standard descriptive statistical methods (such as mean) were calculated, and a comparison of the proportions was performed using a two-tailed Chi squared test. Differences were considered to be significant for a p-value ≤ 0.05 [30].

### Ethical approval

Ethics committee of both hospitals gave approval for publication of this study (Verona Hospital “Protocol PTP49_Italy (Pr numB1207) signed by the secretariat (Dr Anna Fratucello) on benhalf of President Professor Roberto Leone; Roma Hospital approved on 22nd December 2011, signed by the President Professor Maria Grazia Marciani.

## Competing interests

All authors declare no financial or personal relationships with other people or organizations that could inappropriately have influenced (bias) their work.All coauthors have no specific conflict of interests.

## Authors’ contributions

CF and GLC, contributed to the conception of the study, in data analysis and are also involved in drafting the manuscript. CS, MP contributed in acquisition and interpretation of data. All authors approved the final version of the manuscript.
